# Finding gaps in routine TB surveillance activities in Bangladesh

**DOI:** 10.5588/ijtld.21.0624

**Published:** 2022-04-01

**Authors:** A. Allorant, S. Biswas, S. Ahmed, K. E. Wiens, K. E. LeGrand, M. M. Janko, N. J. Henry, W. J. Dangel, A. Watson, B. F. Blacker, H. H. Kyu, J. M. Ross, M. S. Rahman, S. I. Hay, R. C. Reiner

**Affiliations:** 1 Department of Global Health, University of Washington, Seattle, WA; 2 Institute for Health Metrics and Evaluation, Seattle, WA, USA; 3 International Centre for Diarrhoeal Disease Research, Bangladesh (icddr,b), Dhaka, Bangladesh; 4 Department of Epidemiology, Johns Hopkins Bloomberg School of Public Health, Baltimore, MD, USA; 5 Big Data Institute, University of Oxford, Oxford, UK; 6 Department of Health Metrics Sciences, School of Medicine, University of Washington, Seattle, WA, USA; 7 Department of Medicine, Division of Allergy and Infectious Diseases, University of Washington, Seattle, WA, USA

**Keywords:** tuberculosis, tuberculosis prevalence survey, case notification, National Tuberculosis Control Programme, SDG-3, spatial analysis, geospatial modeling, survey methods

## Abstract

**BACKGROUND:**

TB was the leading cause of death from a single infectious pathogen globally between 2014 and 2019. Fine-scale estimates of TB prevalence and case notifications can be combined to guide priority-setting for strengthening routine surveillance activities in high-burden countries. We produce policy-relevant estimates of the TB epidemic at the second administrative unit in Bangladesh.

**METHODS:**

We used a Bayesian spatial framework and the cross-sectional National TB Prevalence Survey from 2015–2016 in Bangladesh to estimate prevalence by district. We used case notifications to calculate prevalence-to-notification ratio, a key metric of under-diagnosis and under-reporting.

**RESULTS:**

TB prevalence rates were highest in the north-eastern districts and ranged from 160 cases per 100,000 (95% uncertainty interval [UI] 80–310) in Jashore to 840 (UI 690–1020) in Sunamganj. Despite moderate prevalence rates, the Rajshahi and Dhaka Divisions presented the highest prevalence-to-notification ratios due to low case notifications. Resolving subnational disparities in case detection could lead to 26,500 additional TB cases (UI 8,500–79,400) notified every year.

**CONCLUSION:**

This study is the first to produce and map subnational estimates of TB prevalence and prevalence-to-notification ratios, which are essential to target prevention and treatment efforts in high-burden settings. Reaching TB cases currently missing from care will be key to ending the TB epidemic.

## Abstract (fr)

**INTRODUCTION:**

La TB était la première cause de décès dus à un agent infectieux unique à l’échelle internationale entre 2014 et 2019. Les estimations fines de la prévalence de la TB et les notifications de cas peuvent être assemblées afin d’établir des priorités permettant de renforcer les activités de surveillance de routine dans les pays à forte prévalence. Nous avons effectué des estimations de l’épidémie de TB pertinentes pour l’élaboration de politiques dans la deuxième unité administrative du Bangladesh.

**MÉTHODES:**

Nous avons utilisé un modèle spatial Bayésien ainsi que l’enquête transversale de prévalence nationale de la TB réalisée au Bangladesh en 2015–2016 pour estimer la prévalence de la maladie par district. Nous avons utilisé les notifications de cas afin de calculer un ratio prévalence-notifications, un indicateur clé du sous-diagnostic et de la sous-notification.

**RÉSULTATS:**

Les taux de prévalence de la TB étaient plus élevés dans les districts du nord-est et variaient de 160 cas pour 100 000 habitants (intervalle d’incertitude à 95% [UI] 80–310) à Jessore à 840 (UI 690–1020) à Sunamganj. Malgré des taux de prévalence modérés, les divisions de Râjshâhî et Dhâkâ avaient les plus forts ratios prévalence-notifications en raison d’une faible notification de cas. En gommant les disparités infranationales en matière de détection de cas, 26 500 cas supplémentaires de TB (UI 8 500–79 400) pourraient être notifiés chaque année.

**CONCLUSION:**

Cette étude est la première à fournir et à cartographier des estimations infranationales de la prévalence de la TB et des ratios prévalencenotifications. Ces estimations sont essentielles afin de cibler les efforts de prévention et de traitement dans un pays à forte prévalence. Réussir à atteindre les cas de TB qui passent actuellement entre les mailles du filet est crucial pour mettre fin à l’épidémie de TB.

With an estimated 1.2 million deaths worldwide, TB was the leading cause of death from a single infectious pathogen in 2019.^1^ Despite ambitious global goals set by the WHO and the United Nations (UN) to reduce the global burden of the TB epidemic, an estimated 10 million people developed TB in 2019. The WHO’s End TB Strategy and UN’s Sustainable Development Goals (SDGs) 3.3 aim to end the global TB epidemic, with targets to reduce TB deaths by 95% and new cases by 90% between 2015 and 2035.^2^,^3^ Achieving these targets will require an unprecedented effort to close the gaps in the TB care cascade, by which only 71% of people who develop TB every year are diagnosed and initiate treatment.

Bangladesh is a high TB burden country according to the WHO. The Global Burden of Diseases, Injuries, and Risk Factors (GBD) Study estimated 216,000 incident cases (95% uncertainty interval [UI] 184,400–250,200) and 29,100 deaths (UI 22,400–40,000) among HIV-negative people in 2019 in Bangladesh.^4^ With an estimated treatment coverage of 81%, approximately one in every five TB case in Bangladesh is missing from care every year. Nationally representative TB prevalence surveys were conducted in Bangladesh in 2007–2009 and 2015–2016 and found important differences in TB prevalence by age, sex, socioeconomic status, and rural/urban status.^5^,^6^ An increasing number of spatial analyses of TB prevalence,^7^–^10^ incidence^11^ or mortality^12^ in high-burden countries have shown high within-country variation.

Prior investigations of the spatial distribution of TB in Bangladesh include two recent studies from the KIT Royal Tropical Institute (Amsterdam, The Netherlands) relying on subnational case notification data collected by Bangladesh National TB Control Programme (NTP).^13^,^14^ Both of these studies supply evidence of extensive gaps in TB notifications of certain districts (second-level administrative divisions) of Bangladesh.

In this study, we illustrate how the growing number of TB prevalence surveys can be used to supply subnational estimates of prevalence in Bangladesh. We analyzed data from the National TB Prevalence Survey (2015–2016), produced district-level estimates and uncertainty intervals accounting for the complex survey design, and calibrated the data to national estimates from the GBD study. There are several methodological challenges associated with estimating TB prevalence subnationally. First, the sampling design, a stratified multistage cluster sampling, needs to be accounted for in the analysis. Second, TB is relatively rare in select districts in Bangladesh, where low case counts can lead to potentially unstable estimates. In this study, we addressed these challenges by adapting a Bayesian spatial hierarchical framework to analyze Bangladesh’s 2015–2016 national survey and produce estimates of prevalence for pulmonary TB in those 15 years and older at fine geographical scales. We also used case notification data collected by Bangladesh’s NTP to estimate district-level prevalence-to-notification ratio, a key metric of the gaps in detection and reporting of new cases. Finally, we conducted a subnational counterfactual analysis, which sets the national prevalence-to-notification ratio as the benchmark for every district, and calculated the number of additional TB cases that could be notified by resolving subnational inequalities. Achieving the End TB Strategy and SDG 3.3 will require resources and interventions targeting locations with large numbers of TB cases that are undiagnosed or are lacking treatment coverage. This study shows how local estimates of TB prevalence and prevalence-to-notification ratio can be combined to find areas in greatest need of enhanced access to diagnosis and treatment services.

## METHODS

### Data

**Figure 1:**
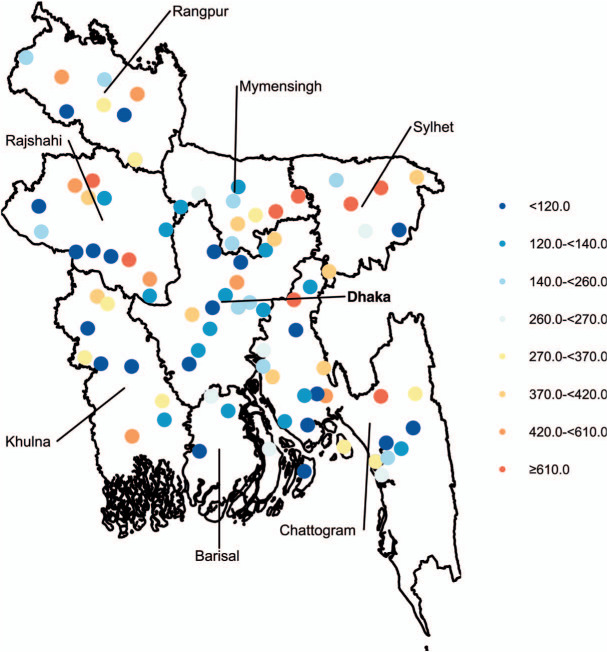
Distribution of surveyed clusters across Bangladesh’s divisions and observed prevalence of TB at these clusters.

Bangladesh’s National TB Prevalence Survey was cross-sectional and used a multi-stage cluster sampling method. Data were collected across Bangladesh (Figure 1) from any person aged ≥15 years, living in one of the 125 clusters sampled (46 urban and 79 rural) between March 2015 and April 2016. Case definition included Xpert^®^ MTB/RIF (Cepheid, Sunnyvale, CA, USA) and/or culture-confirmed cases; smear-positive subjects not confirmed using Xpert were excluded for the estimation of prevalence. There were 98,710 participants in the study, including 20,594 screening positive to either symptom or chest X-ray; two sputum specimens were collected from each of the 20,010 individuals. A total of 278 bacteriologically confirmed cases were considered as study cases, including 108 smear-positive and 170 smear-negative cases. Specific details about the survey complex design and findings are presented extensively elsewhere.^9^ The TB notification data reported by each of the 64 districts of Bangladesh were collected from the NTP. After assessing the consistency of new cases reported to the NTP (see Supplementary Methods 1.4), we used the new and relapse pulmonary TB cases by district in 2016 to calculate a district-level prevalence-to-notification ratio.

### Statistical analysis

TB prevalence was estimated using a small area estimation approach, which is a spatial extension of the classic Fay and Herriot approach.^15^,^16^ First, the survey design was accounted for by directly calculating the Horvitz-Thompson estimator of TB prevalence for each district using the design weights (see Supplementary Methods 1.1.1). Second, the logit transformation of the true district prevalence was modeled as a linear function of covariates and spatially structured random effects. We used rigorous selection techniques to identify the best performing model, which included average temperature as the only covariate (see Supplementary Methods 1.1.3). Of the 64 districts of Bangladesh, 19 contain no sampled clusters. In these locations, our hierarchical approach drew strength from spatially adjacent districts and modeled covariates to predict TB prevalence. Predicted prevalence estimates were divided by the total number of pulmonary cases in those aged ≥15 years notified for the corresponding district in 2016. We conducted a counterfactual analysis in which districts with a prevalence-to-notification ratio above the national average prevalence-to-notification ratio were set to the national average. We used the counterfactual analysis to estimate the number of additional TB cases that could be notified by the NTP every year, if every district reduced its prevalence-to-notification ratio to at least the national average. The national prevalence-to-notification ratio, which was 2.8 in 2016, was used as a benchmark.

### Ethics approval

Ethics approval was not required for this study, as no data were collected for the purposes of this study.

## RESULTS

### District-level estimates of TB prevalence

**Figure 2:**
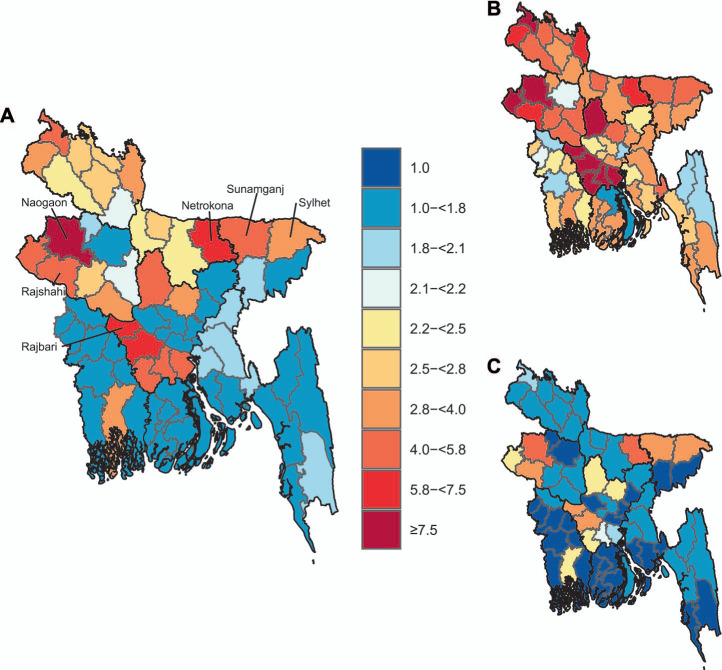
A) Estimated mean TB prevalence-to-notification ratio, and B) 97.5^th^, and C) 2.5^th^ percentiles.

**Figure 3:**
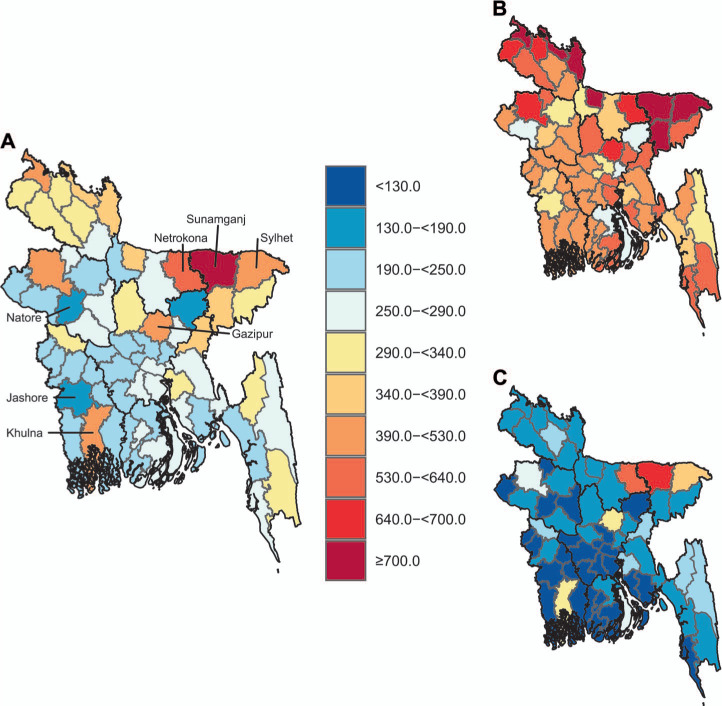
A) Estimated TB mean prevalence (per 100,000), and B) 97.5^th^, and C) 2.5^th^ percentiles.

District-level estimates of TB prevalence revealed a clear spatial pattern in TB prevalence, with higher rates in northern and eastern districts than south-western districts. North-eastern districts displayed the highest TB prevalence rates (Figure 2). Specifically, Sunamganj, with 840 cases/100,000 (95% UI 690–1020), Netrokona with 640 cases/100,000 (UI 580–690), and Sylhet with 520 cases/100,000 (UI 370–710) were the three districts with the highest prevalence rates nationally (Supplementary Table S4 presents all district-level prevalence estimates). Predicted TB prevalence overall varied significantly by district: Sunamganj, Netrokona, and Sylhet, the three districts with prevalence rates in the 95^th^ percentile nationally, had prevalence rates three times higher than districts in the 5^th^ percentile, such as Natore, with 180 cases/100,000 (UI 90–340), and Jashore, with 160 cases/100,000 (UI 80–310). Uncertainty around these estimates was high in some districts; however, five districts – Sunamganj, Netrokona, Sylhet, Gazipur and Khulna – displayed prevalence rates significantly higher than the national average, 287/100,000 (Figure 3).

### District-level estimates of TB prevalence-to-notification ratio

Prevalence-to-notification ratios were larger than the national average (2.8) in northern and central districts. For example, Naogaon (7.9, 95% UI 5.3–12.0), Rajbari (6.7, 95% UI 3.2–14.1), Faridpur (6.1, 95% UI 2.9–12.8), and Netrokona (5.9, 95% UI 5.4–6.5) display prevalence-to-notification ratios in the 90^th^ percentile nationally.

Despite low TB prevalence rates, most districts of the Rajshahi and Dhaka Divisions showed a large prevalence-to-notification ratio. The districts of Rajbari and Faridpur had the second and third largest prevalence-to-notification ratios nationally, despite moderate TB prevalence rates, 240/100,000 (95% UI 110–500) and 240/100,000 (UI 120–510), respectively. These rates can be explained by strikingly low case notification rates, 31 and 36/100,000, in Rajbari and Faridpur, respectively. More generally, 12 out of the 13 districts in the bottom 20% of case notification rates nationally were in the Rajshahi and Dhaka Divisions, which seems to account for the cluster of high prevalence-to-notification ratios in the South-west districts of these two divisions. With a country mean prevalence-to-notification ratio of 2.8, the NTP can detect around one-third of all potential TB cases nationally.^9^

**Figure 4:**
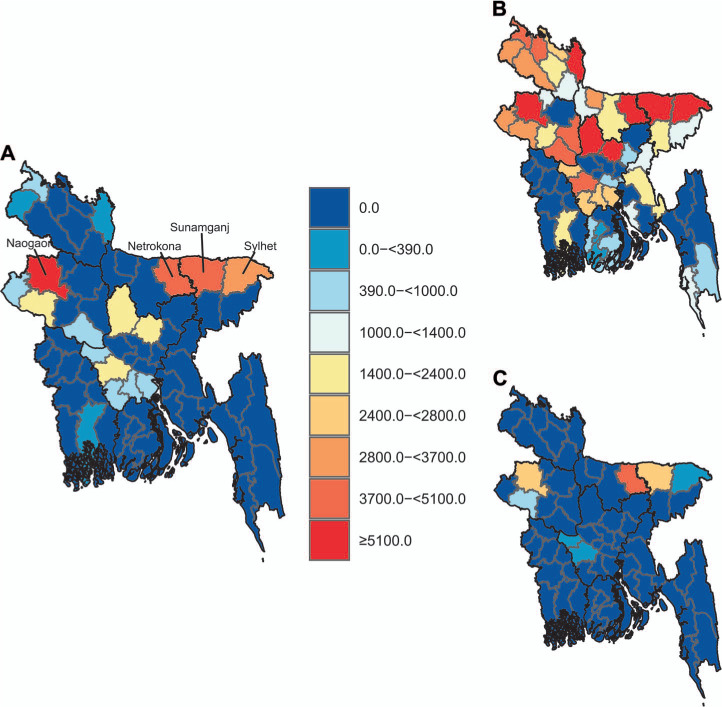
Estimated mean TB additional cases that could be detected if every district achieved at least A) the national prevalence-to-notification ratio, and B) 97.5^th^ and C) 2.5^th^ percentiles.

Assuming the NTP could strengthen routine surveillance and case-detection strategies such that every district reduced its prevalence-to-notification ratio to at least the national average of 2.8, we estimated that an additional 30,800 individuals (95% UI 10,000–84,500) with TB could be diagnosed, notified, and started on life-saving TB treatment. The districts of Naogaon, Sunamganj, Netrokona, and Sylhet accounted for more than half of this reservoir of potential cases, with 15,900 additional notified cases (UI 7,900–27,400) (Figure 4).

## DISCUSSION

This study is, to our knowledge, the first nationwide study investigating within-country variations in TB prevalence while accounting for a complex survey design. Our findings suggest that despite considerable progress made nationally in reducing TB incidence, significant inequalities in the TB prevalence rates and the prevalence-to-notification ratio persist between districts of Bangladesh.

First, prevalence estimates were highest in the northern and north-eastern districts of Bangladesh, with rates up to 2.4 times the national average. In the absence of reliable local indicators on TB incidence, subnational TB prevalence estimates are essential to target prevention efforts in areas with higher ongoing transmission. Intensive TB control interventions targeted at high burden areas could effectively reduce the high prevalence rates highlighted in this study. Interestingly, these north-eastern districts additionally display a higher-than-average proportion of relapse among new cases (see Supplementary Figure S4; Supplementary Methods 2), an indicator of program performance in starting and keeping TB patients on treatment. Geographically distant from Dhaka, the populations living in these mostly rural districts may experience an increased time and financial burden for taking part in the demanding 6–12-month TB treatment course dispensed at health facilities.^17^ Second, our joint study of prevalence and notification data expanded the body of evidence of significant under-reporting and under-diagnosis of TB cases in Bangladesh. We drew from prevalence and case notification data to expand previous evidence of under-reporting and under-diagnosis in divisions where TB prevalence rates are moderate, but case notifications are strikingly low, and in districts, where TB prevalence rates are high. The 2017 Service Provision Assessment, a large-scale facility survey, revealed low-levels of TB diagnostic and treatment services’ availability throughout Bangladesh. We derived from this data source estimates of district-level diagnostic and treatment services’ availability (see Supplementary Figure S6) and compared it to our district-level estimates of prevalence-to-notification ratio. We noted that estimated prevalence-to-notification tended to be lower in districts where TB service availability was high, suggesting that disparities in availability of TB services could explain some of the differences in prevalence-to-notification ratio between districts. Third, we showed the potential impact of resolving subnational heterogeneity in surveillance and TB control activities and estimated the number of “missing” TB cases by district. Each missed case represents a missed opportunity to notify and treat people developing TB, which in turn hinders progress towards ending the TB epidemic. In high-risk areas, strategies to identify people with active disease can supplement routine surveillance activities to enhance case detection.^18^

There are several limitations to this study. First, while our approach enabled us to depict the complex picture of the heterogeneous spatial distribution of TB across Bangladesh, it does not provide further information on temporal trends. The first national prevalence survey in Bangladesh conducted in 2007–2009 used a different methodology leading to much lower estimates of TB prevalence (79.4/100,000), preventing direct comparisons with the 2015–2016 survey. Second, our study is restricted to individuals 15 years and older as defined by the inclusion criteria of the national survey. However, a previous study showed that the pediatric TB epidemic in Bangladesh is substantial;^19^ studying spatial patterns in people under age 15 would supplement our work. Third, our analysis did not differentiate between drug-susceptible TB, multidrug-resistant TB (MDR-TB), and extensively drug-resistant TB, despite distinct policy implications; in Bangladesh, a high MDR-TB burden country according to the WHO, fine-scale estimates of MDR-TB could guide prioritized access to drug susceptibility testing and second-line treatment regimens.^20^ Fourth, despite important differences in TB prevalence, access to TB services and TB mortality, between rural and urban areas, we did not stratify our analysis on rural/urban residence, as this would have led to estimates with large uncertainty. Fifth, while our study reinforced earlier evidence of substantial gaps in case notifications in many districts of Bangladesh, our interpretation lacked the tools to disentangle the causes of these gaps – treatment-seeking behaviors, accessibility of services, or reporting completeness – and their respective importance merits further investigation.^21^,^22^ Sixth, in the absence of subnational prevalence data for well-established TB risk factors, including smoking, alcohol use, and diabetes, we had a limited number of auxiliary variables that could be used to enhance the precision of our prevalence estimates. We estimated the prevalence of overcrowding at the district level, using a Bayesian spatial model and Bangladesh DHS 2007, 2011, and 2014 survey data. This constructed exposure was included, along with average daily temperature, in the best model according to one model selection procedure (conditional predictive ordinate). Finally, our analysis relies on data from 2016, and the disruptions in TB services caused by COVID-19 in Bangladesh may have changed the geographical patterns described in the present work.^23^

We envision several directions to build on this work. First, the results could be leveraged to improve existing methods that derive incidence from prevalence surveys by assuming a fixed relationship between prevalence, incidence, and duration of disease. Duration of disease is longer in undiagnosed cases than in treated patients. Therefore, the relationship between incidence and prevalence may be distorted in areas with large numbers of TB cases missing from care. Second, employing an integrated modeling approaches linking transmission dynamics and health systems has the potential to provide invaluable insights into the understanding of the optimal delivery of TB services.^24^ Subnational estimates of TB prevalence and case notification rates, along with local estimates of access to quality TB services from health facility survey data, could be used as inputs to represent spatial heterogeneity in these mathematical models.^25^ Finally, the method presented in this study could be replicated in other high-priority settings with case notification counts and recent prevalence surveys, such as the Philippines (2016), Vietnam (2017), Myanmar and Namibia (2018), South Africa (2019), and India (2021).

## CONCLUSIONS

In conclusion, our study shows that even when countries such as Bangladesh have achieved considerable progress in case notifications and treatment coverage, substantial within-country inequalities on key indicators of the TB epidemic are still observed. Our analysis suggests that the large numbers of people who develop TB and are missing from care each year are concentrated in select districts. Enhanced routine surveillance activities along with active case-finding strategies will be key to reach, diagnose, and treat people with TB in these areas that likely face barriers in accessing testing and treatment services. Ending TB in Bangladesh by 2035, in alignment with the WHO and UN strategies, will require closing the gaps in diagnostic, prevention, and treatment efforts. Subnational estimates of TB prevalence and prevalence-to-notification ratios can guide these efforts, help develop tailored strategies, and ensure that no population is left behind in the fight against TB.
